# Supplementary material for: Mpox due to monkeypox virus clade Ia infection detected outside of Africa: a case study
Source: Commun Med (Lond). 2025 Nov 22;5:537. doi: 10.1038/s43856-025-01247-1 (PMC12748866; doi:10.1038/s43856-025-01247-1)
Supplement: Supplementary file 2 — Description of Additional Supplementary files [file 43856_2025_1247_MOESM2_ESM.docx]

**Description of Additional Supplementary Files**

Supplementary Data 1: Custom primers added to sequencing workflow. Primers were designed using Primer3 to recover amplicons that dropped out when using the MBS primer pools. Each row lists the target amplicon, the forward and reverse primer sequences and the expected amplicon product size (bp).

Supplementary Data 2: Details of the 182 published sequences that clustered with the new Irish sequence in the 2024 Kinshasa Outbreak cluster.

Supplementary Data 3. Details of all 61 single nucleotide substitutions identified in the Illumina-sequenced MPXV Clade Ia Irish genome, mapped to the reference genome
